# Date fruit melanin is primarily based on (−)-epicatechin proanthocyanidin oligomers

**DOI:** 10.1038/s41598-024-55467-x

**Published:** 2024-02-28

**Authors:** Muneeba Zubair Alam, Clinton Emeka Okonkwo, João P. Cachaneski-Lopes, Carlos F. O. Graeff, Augusto Batagin-Neto, Saeed Tariq, Sabu Varghese, Matthew J. O’Connor, Abuzar E. Albadri, J. Beau W. Webber, Mohammed Tarique, Mutamed Ayyash, Afaf Kamal-Eldin

**Affiliations:** 1 Department of Food Science, College of Agriculture and Veterinary Medicine, United Arab Emirates University, P.O. Box: 15551, Al-Ain, United Arab Emirates; 2 Postgraduate Program in Materials Science and Technology (POSMAT), São Paulo State University (UNESP), Bauru, SP Brazil; 3 Department of Physics, School of Sciences, São Paulo State University (UNESP), Bauru, SP Brazil; 4 Institute of Sciences and Engineering, São Paulo State University (UNESP), Itapeva, SP Brazil; 5 Department of Anatomy, College of Medicine and Health Sciences, United Arab Emirates University, Al Ain, United Arab Emirates; 6 Core Technology Platforms, New York University Abu Dhabi, 129188 Abu Dhabi, United Arab Emirates; 7 Department of Chemistry, College of Science, Qassim University, 51452 Buraidah, Saudi Arabia; 8 Lab-Tools Ltd., Marlowe Innovation Centre, Marlowe Way, Ramsgate, CT12 6FA UK; 9 National Water and Energy Center (NWEC), United Arab Emirates University, P.O. Box: 15551, Al-Ain, United Arab Emirates

**Keywords:** Date fruit, *Phoenix dactylifera* L., Melanin, Proanthocyanidins, (−)-Epicatechin, Electron microscopy, Molecular modelling, NMR spectroscopy

## Abstract

Plant-based melanin seems to be abundant, but it did not receive scientific attention despite its importance in plant biology and medicinal applications, e.g. photoprotection, radical scavenging, antimicrobial properties, etc. Date fruit melanin (DM) has complex, graphene-like, polymeric structure that needs characterization to understand its molecular properties and potential applications. This study provides the first investigation of the possible molecular composition of DM. High performance size-exclusion chromatography (HPSEC) suggested that DM contains oligomeric structures (569–3236 Da) and transmission electron microscopy (TEM) showed agglomeration of these structures in granules of low total porosity (10–1000 Å). Nuclear magnetic resonance (NMR) spectroscopy provided evidence for the presence of oligomeric proanthocyanidins and electron paramagnetic resonance (EPR) spectroscopy revealed a g-factor in the range 2.0034–2.005. Density functional theory (DFT) calculations suggested that the EPR signals can be associated with oligomeric proanthocyanidin structures having 4 and above molecular units of (−)-epicatechin. The discovery of edible melanin in date fruits and its characterization are expected to open a new area of research on its significance to nutritional and sensory characteristics of plant-based foods.

## Introduction

Plant foods (including cereals, fruits, vegetables, legumes, and seeds) contain a wide range of antioxidant phenolic compounds^1^. These compounds can be divided into soluble forms, based on their solubility and extractability in organic solvents with or without acid or base hydrolysis, and insoluble bound forms, which remain in the pellet together with fiber and other residues after extraction of the soluble forms^2,3^. Owing to their complex and high molecular weight (Mw) structures, there are limited studies on the insoluble phenolic compounds. Melanin pigments have been identified as insoluble phenolic constituents in some plant foods^4^.

We have identified three types of phenolic compounds in date fruits, i.e. soluble, hydrolysable, and polymeric insoluble forms^5^. The soluble phenolic compounds can be extracted by organic solvents and are mainly concentrated in cell vacuoles. The hydrolysable forms can be extracted with organic solvents after alkaline hydrolysis and represent phenolic compounds that are bound to the cell walls via ester linkages^6^. Date fruits also contain (−)-epicatechin-based oligomeric proanthocyanins (degree of polymerization 7–33) that are mainly concentrated in specialized tannin cells^7–9^. In addition, date fruits contain high percentages of insoluble polymeric lignin^10^ and melanin^11^. Melanin pigments may result from enzymatic browning that occur during fruit development and storage^12^. Postharvest browning of dates is primarily attributed to the oxidation of phenolic compounds by polyphenol oxidase (PPO) and/or peroxidase (POD)^13^. PPO can be involved in the *ortho*-hydroxylation of monophenols to diphenols, followed by their oxidation to quinones having a high tendency to polymerize into melanin^14^. The differences in the chemical structures of the wide range of phenolic compounds in date fruits limits the applicability of the “total antioxidant activity” methods, which are only able to assess the soluble phenolic compounds^5^.

Melanin refers to a unique group of black and brown phenolic pigments present in animals, fungi, bacteria, and plants. It has a high Mw and is thermally stable, is insoluble in water, aqueous acids, and common organic solvents, and is resistant to reducing agents and light^15^. Melanin has various beneficial properties, including antitumor, antioxidant, radiation resistant, anti-inflammatory, antimicrobial, neuroprotective, nanoparticle synthesizing, liver protection, radioactive residue remediation, and digestive system protection effects^16^. Owing to its excellent biodegradability and biocompatibility, melanin can be used for several biomedical applications, including drug delivery, photothermal therapy, and bioimaging systems^17^. Moreover, melanin is useful in the cosmetic industry, e.g. in the synthesis of products such as sunscreen and hair dyes^18^.

We have previously reported on the presence of high concentrations of melanin in date fruits^19^ but the chemical nature of this melanin was not elucidated. The current study aimed to fill this gap by investigating the chemical nature of DM by employing a combination of microscopic, chromatographic, and spectroscopic techniques aided by computational modeling. The key finding of the present study is that the DM is based on (−)-epicatechin proanthocyanin (PACs) oligomers.

## Materials and methods

### Date palm fruits

Fruits of four Emirati date cultivars (Dabbas, Fard, Khalas, Neghal) obtained from Al Foah Dates Factory (Al Saad, Abu Dhabi, UAE) and two cultivars from Saudi Arabia (Ajwa and Safawi) purchased from the market (Al Ain, Abu Dhabi, UAE) were used in this study. The fruits were collected at the fully mature Tamar stage and stored at 4 °C until analysis. All the plant experiments followed relevant institutional, national, and international guidelines and legislation.

### Chemicals

4-Dimethylamino-cinnamaldehyde (DMACA), dimethyl sulfoxide (DMSO), 2,2-diphenyl-1-picrylhydrazyl (DPPH), formic acid, melanin from *Sepia officinalis*, n-butanol, phosphate buffer, potassium ferricyanide, potassium permanganate, potassium persulfate, potassium phosphate buffer, sodium borate buffer, sodium carbonate, sodium hydroxide, sodium potassium tartrate, and sulfuric acid was purchased from Sigma Chemical Company (St. Louis, Missouri, USA). Analytical-grade acetic acid, acetone, acetonitrile, chloroform, ethanol, ethyl acetate, hydrochloric acid, and methanol were purchased from Honeywell (Seelze, Hanover, Germany). Deuterated dimethyl sulfoxide (DMSO), used in NMR studies, was purchased from Cambridge Isotope Laboratories (Tewksbury, MA, USA). All other chemicals and reagents were of analytical grade and were used without purification.

### Melanin extraction and characterization strategy

**Figure 1:**
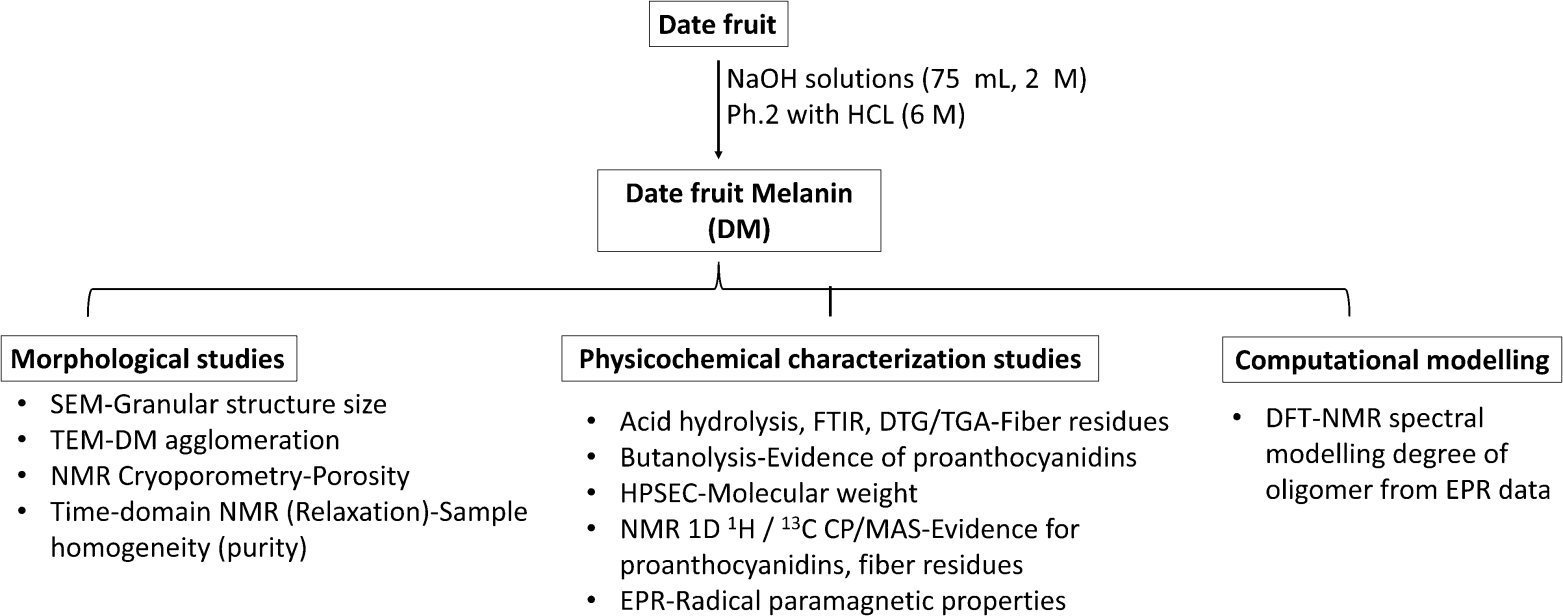
Experimental strategy. SEM: scanning electron microscopy; TEM: transmission electron microscope; NMR: nuclear magnetic resonance; FTIR: Fourier transform infrared spectroscopy; DTG: derivative thermal gravimetric analysis; TGA: thermogravimetric analysis; HPSEC: high performance size exclusion chromatography; 1D ^1^H NMR: one dimensional proton nuclear magnetic resonance; ^13^C CP/MAS: carbon-13 cross polarization magic angle spinning; EPR: electron paramagnetic resonance; DFT: density functional theory.

The experimental strategy followed to reveal the nature of DM included morphological, physicochemical, and computational studies is shown in Fig. 1 and explained in details below. Extraction of DM was carried out using 2M NaOH as described before^19^. Thereafter, the extracted melanin was subjected to acid hydrolysis (6 M HCl, 1:10 w/v, 110 °C, 8 h) to investigate the presence residual fiber components. The acid hydrolysis was followed by filtration through a 11-µm membrane filter to collect the solid residue, which was washed with distilled water to neutral pH. The residue was further washed with organic solvents (ethanol, acetone, and chloroform), and then freeze-dried.

### Morphological studies of date fruit melanin

DM samples were observed in the secondary electron imaging mode using an analytical scanning electron microscope (SEM, JEOL JSM-6010PLUS/LA, Tokyo, Japan). The samples were sputter-coated in gold, mounted onto aluminum stubs painted with silver to act as an adhesive conductor, and examined under low vacuum with a power of 20 kV^19^. For transmission electron microscopy (TEM-FEI/Tecnai Spirit G2 Bio Twin from Eindhoven, Netherlands), the melanin samples were deposited onto a 200-mesh formvar/carbon-coated copper grid. Pore size and time-domain NMR relaxation studies were conducted using CryoP4 NMR cryoporometry probe and Mk3 NMR spectrometer (Lab-Tools, Nano-Science, Kent, UK), as previously described^20^.

### Physio-chemical characterization of date fruit melanin

DM samples, with and without acid treatment, were analyzed at room temperature via attenuated total reflectance spectroscopy (ATR-FTIR, Perkin-Elmer Inc., Norwalk, CT, USA) within the spectral region of 4000–400 cm^−1^^19^. Thermogravimetric analysis (TGA) was performed using a Netsch STA409 EP thermogravimetric analyzer (Selb, Germany) by heating the DM samples (10 mg) under a nitrogen atmosphere between 20 and 700 °C at a heating rate of 10 °C min^−1^. The weight loss (%) during TGA and derivative weight change (derivative thermogravimetric analysis (DTG)) at increasing temperatures were continuously recorded and plotted^21^.

Butanolysis, followed by scanning UV absorption, was performed to test for the presence of proanthocyanidins in the crude melanin. Crude melanin (10 mg) was mixed with n-butanol (20 mL) and concentrated sulfuric acid (0.3 mL) and heated in a water bath at 100 °C for 5 h^22^. The colored supernatant was scanned using a UV spectrophotometer at the wavelength range of 200–900 nm (Multiskan GO, Thermo Fisher Scientific, Finland). The molecular weight (Mw) of DM was determined by high performance size-exclusion chromatography (HPSEC) on a Shimadzu ystem (Kyoto, Japan) fitted with a refractive index detector (RID-20A). The samples were run through a 0.22 μm syringe filters before injection into the chromatographic column (Shim-pack GPC-802, Shodex^®^), maintained with the detector at 40°C. The samples were eluted with distilled water at a rate of 1 mL/min and the Mw was calculated by constructing a calibration curve using different pullulan standards (Showa DENKO, Tokyo, Japan) having Mw ranging from 0.342 to 800 kDa^23^.

1D ^1^H NMR spectra were obtained using a Bruker Avance-HD 500 MHz spectrometer (Bruker Biospin Gbh, Germany) operated with a static field of 11.7 T using a PA BBF-H-D ZSP probe. The samples were dissolved in deuterated DMSO and a standard proton NMR pulse sequence was applied (16 scans). The spectra were processed using the MestReNova software (Mestrelab Research, A Coruna, Spain). To enhance the signal to noise ratio, the line broadening function was increased to 1.5 Hz and the baseline was corrected using the full auto spline function. Magic angle spinning (MAS) solid-state NMR experiments were performed using 600 MHz Bruker Aeon-HD spectrometer (Bruker BioSpin GmbH, Germany) operating within a static field of 14.1 T using a 4.0 mm MAS probe. The samples were packed into 4.0 mm zirconia rotors and were spun at a MAS frequency of 14kHz. Then ^1^H-^13^C cross-polarization (CP/MAS) experiments were performed using a standard linearly ramped CP pulse sequence. Further, ^13^C chemical shifts were externally referenced to the adamantane CH_2_ signal at 38.48 ppm. NMR data were processed using TopSpin software (Bruker BioSpin GmbH, Germany).

EPR measurements were performed in solid-state using an X-band spectrometer MiniScope MS300 (Magnettech, Berlin, Germany). Microwave frequency was measured using an Agilent Frequency Counter 53181A RF at a microwave power ranging from 0.10 to 50.12 mW. DPPH (g = 2.0036) was used for g-factor calibration^24^. Simulation of the EPR spectra was performed using EasySpin (v.5.2.35, https://easyspin.org/)^25^ with the aid of pepper routine considering Lorentzian line shapes.

### Computational modeling based on density functional theory (DFT) calculations

**Figure 2:**
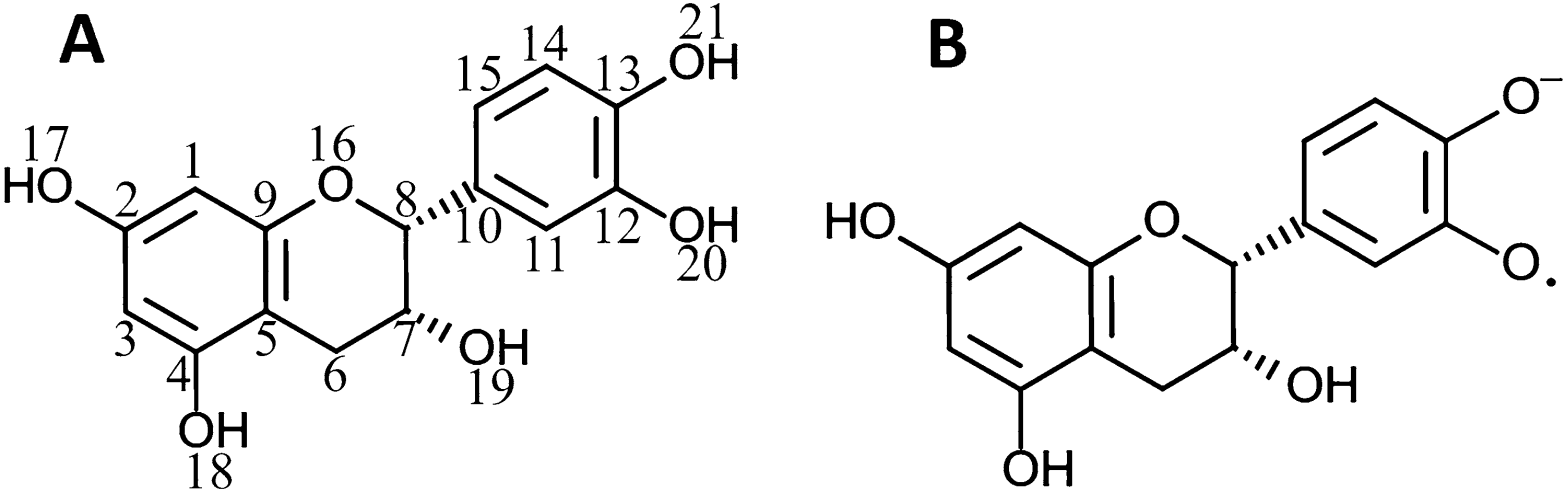
Chemical structures of (**A**) (−)-epicatechin and (**B**) (−)-epicatechin radical anion.

DFT calculations were used to investigate distinct structures of (−)-epicatechin oligomers, enabling better interpretation of the experimental results. All structures were designed using the GaussView computational package (Gaussian.com)^26^ and were fully optimized within the DFT framework considering a B3LYP exchange–correlation hybrid functional and 6-311G(d,p) basis set for all atoms. Figure 2 depicts the structures of (−)-epicatechin (a) and its radical (b) that were considered for DFT calculations^27^.

To establish representative polymer models, oligomeric structures with two, three, four and five units were designed by linking adjacent blocks through 1–6 connections (Fig. 2). NMR chemical shifts (σ_i_) were estimated via DFT calculations and converted into relative chemical shifts (δ_i_) considering trimethyl silane (TMS) as a reference (δ_i_ = σ_TMS_ − σ_i_)^28^. The geometry optimization and NMR calculations were conducted using the DFT/B3LYP/6-311G(d,p) approach. Scaling factors (SF) were employed to compare the theoretical and experimental values^29^. A straightforward method was used, where the SF is defined as SF = δ_exp_/δ_i_, with δ_exp_ representing the experimental signal value, and δ_i_ denoting the respective relative chemical shift. The corrected chemical shifts were estimated as δ_corrected_ = δ_i_ × SF.

EPR parameters were calculated using Orca computational package version 4.0.1.2^30^, whereas the remaining calculations were performed using the Gaussian 16 computational package (Gaussian 16 Revision A.03. Gaussian Inc. Wallingford CT). Monomeric structures were used to validate the theoretical approach through comparison with literature data. Geometry optimization and chemical shifts evaluation of the structures were performed using DMSO as a solvent. EPR calculations were conducted *in vacuo* for all structures and band gap variation was evaluated to identify saturation effects (i.e. *ΔE*_*gap*_ < *k*_*B*_*T*_*300*_ ~ *0.025 eV*). The presence of solvents was simulated using the polarizable continuum model (PCM) method^31^.

## Results and discussion

### Morphological studies of date fruit melanin

**Figure 3:**
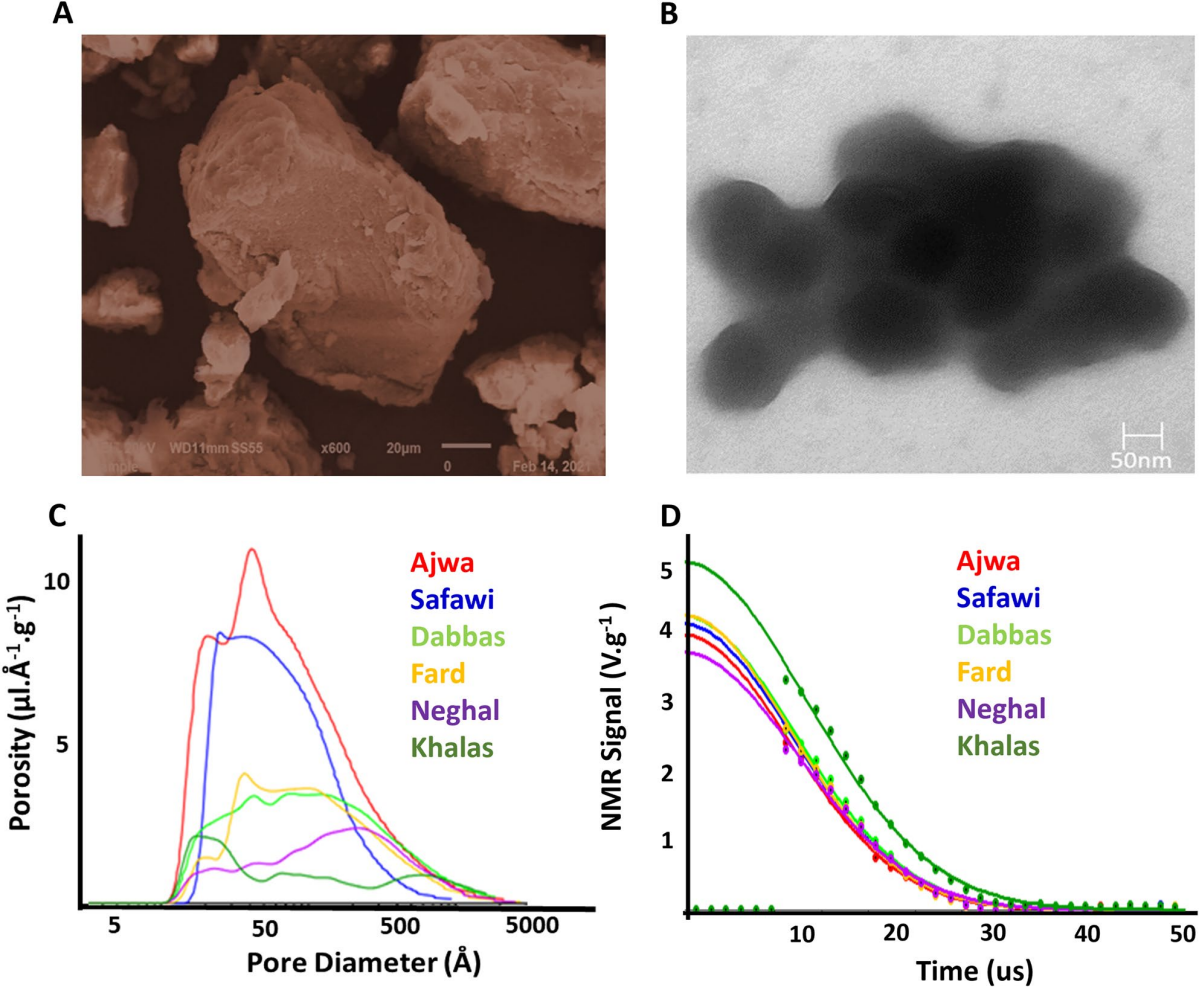
Morphology of the DM granules (**A**) colored scanning electron microscopic (SEM) images of DM particles (×600 at 20 µm) and (**B**) transmission electron micrograph (TEM) at 50 nm extracted from Dabbas cultivar, (**C**) pore distribution graphs for the DM extracted from 6 cultivars obtained by NMR cryoporometry, and (**D**) NMR relaxation times (T2).

The morphologies of the melanin samples extracted from 6 date fruit cultivars were comparable; therefor, SEM and TEM micrographs of the Dabbas cultivar were presented as representative. The SEM micrographs (Fig. 3A) revealed that DM granules consist of amorphous graphene-like granular structures with irregular shapes and variable sizes having dimensions ranging from 43 to 350 µm^19^. It was shown that the morphology and size of melanin granules extracted from various fungal and bacterial sources vary with the source and method of extraction^4^. TEM micrographs (Fig. 3B) indicated that DM is formed via the agglomeration of many aggregates, consistent with previous findings of sepia ink melanin^32^. NMR cryoporometry revealed that the extracted DM granules have low total porosity with pore size varying from 10 to 1000 Å (Fig. 3C). The differences in samples porosity are presumably affected by the preparation method, particularly the acid precipitation step leading to the agglomeration and formation of the granules. Time-domain NMR was used to assess the molecular environment and dynamics of the samples^33^. The relaxation time, T2, which refers to the amplitude decay of the NMR signal over time due to spin–spin relaxation processes, is an essential component that provides information reflecting sample homogeneity. The samples exhibited only one peak in the T2 spectrum (Fig. 3D) suggesting high homogeneity.

### Physio-chemical characterization of date fruit melanin

**Figure 4:**
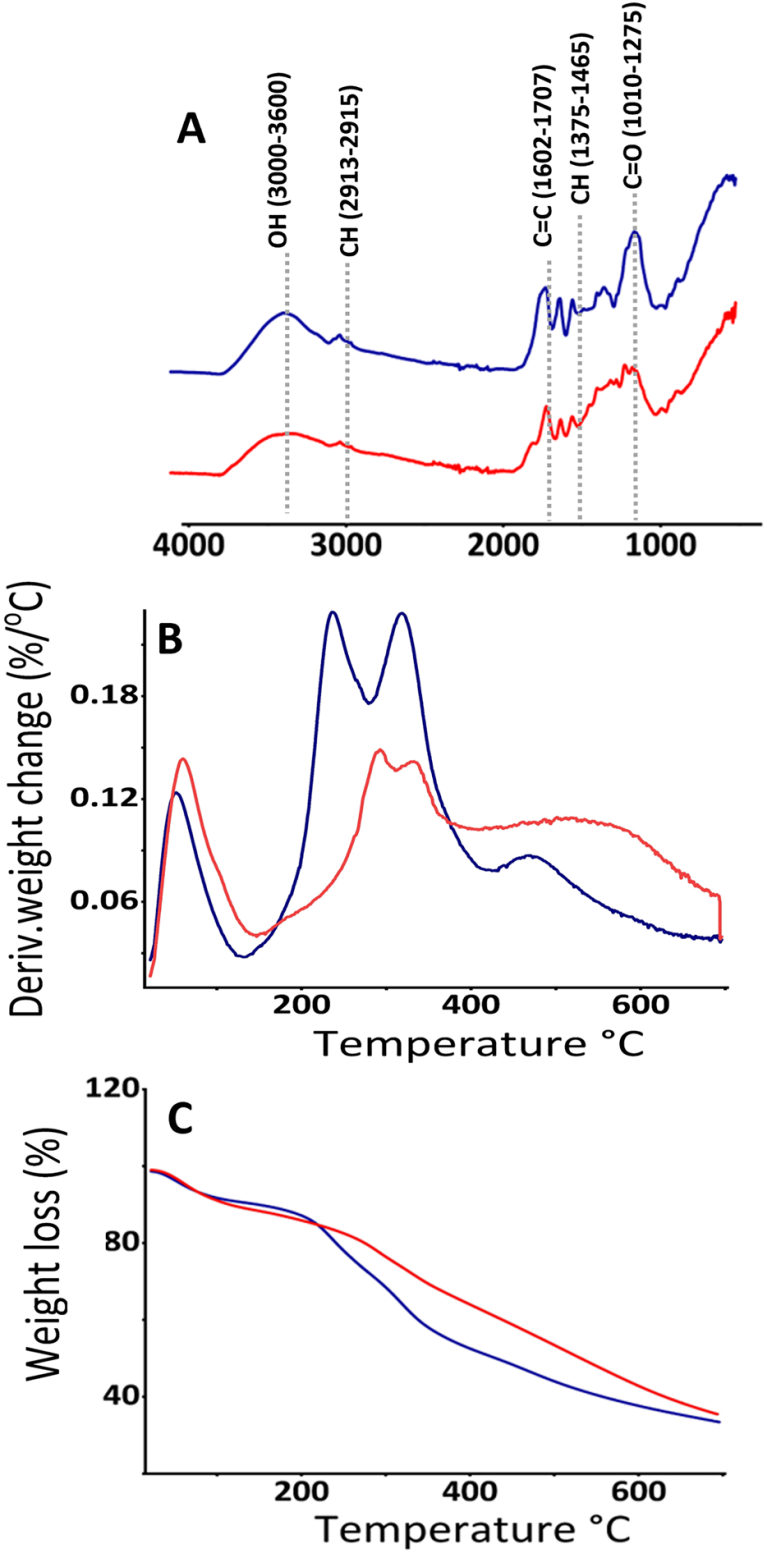
Characteristics of melanin samples before and after acid hydrolysis (6 M HCl) (**A**) Fourier transform infrared spectroscopy (FTIR), (**B**) derivative thermogravimetric analysis (DTG), (**C**) thermogravimetric analysis (TGA). Samples before and after acid hydrolysis are shown in blue and red, respectively.

Acid treatment was applied to the DM to remove other alkali-soluble fiber components, such as cellulose, hemicellulose, and lignin that may have been co-extracted with the melanin. FTIR spectroscopic analysis of the DM samples revealed comparable peak patterns before and after acid treatment (Fig. 4A). The broadband absorption at approximately 3235–3281 cm^−1^ corresponds to the stretching vibration of OH groups^34^. The peaks observed in the range of 2913–2915 cm^−1^ were assigned to stretching vibration of the CH groups, the characteristic band at 1602 cm^−1^ corresponds to the vibrations of the aromatic ring C=C or symmetric stretching of COO groups, the band at 1432–1437 cm^−1^ represents the CH_2_–CH_3_ bending, and the peak at approximately 1010–1275 cm^−1^ indicates the stretching vibration of C=O^32^. The acid treatment partially decreased the C=O stretching vibration band suggesting that the extracted melanin is a crude preparation that may contain some fiber constituents.

TGA and DTG analyses revealed three and two regions of thermal degradation for DM samples before and after acid treatment (Fig. 4B,C), respectively, consistent with the findings on black garlic and sepia ink melanin^32^. The first region accounted for 15% weight loss (at 225 °C) before and 19% weight loss (at 269 °C) after acid treatment indicating an increased weight loss (%) and shift of the degradation temperature to a higher value. The second region, accounting for 28% weight loss (at 368 °C) before acid treatment and 45% weight loss (at 700 °C) after acid treatment, suggest that the extracted DM was impure. A distant third region (368–700 °C), accounting for 22% weight loss, was visible in the untreated but not in the acid-treated samples. Further studies should consider the purity of alkali-extracted plant melanin and any possible contents of fiber constituents.

**Figure 5:**
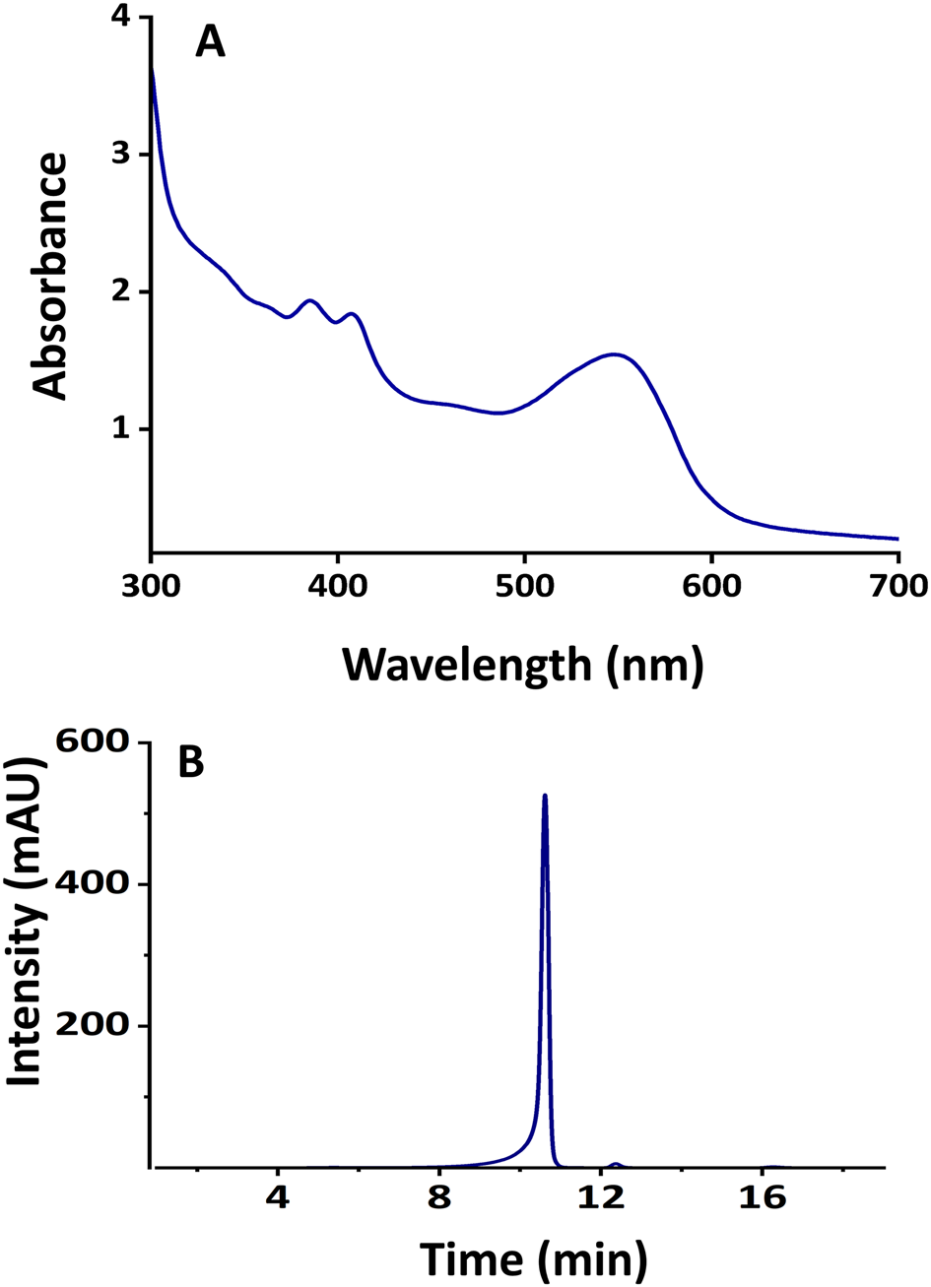
Characteristic of melanin (**A**) spectrum of cyanidin from acid butanol assay, (**B**) typical size exclusion chromatogram of DM.

Treatment of the DM samples with acid butanol resulted in the characteristic color formation of cyanidin suggesting that this melanin is composed of proanthocyanins (PAC) (Fig. 5A). HPSEC suggested that the molecular weight of the extracted DM ranged 569–3236 kDa (Fig. 5B), which corresponds to *ca* 2–11 (−)-epicatechin monomeric units (Mw 290 Da/unit)., which is comparable to the Mw of the melanin extracted from apricot kernel (0.56–2.5 kDa)^35^. Melanin samples were dissolved in alkali during HPSEC, which may facilitate the Mw estimation by breaking π–π stacking.

**Figure 6:**
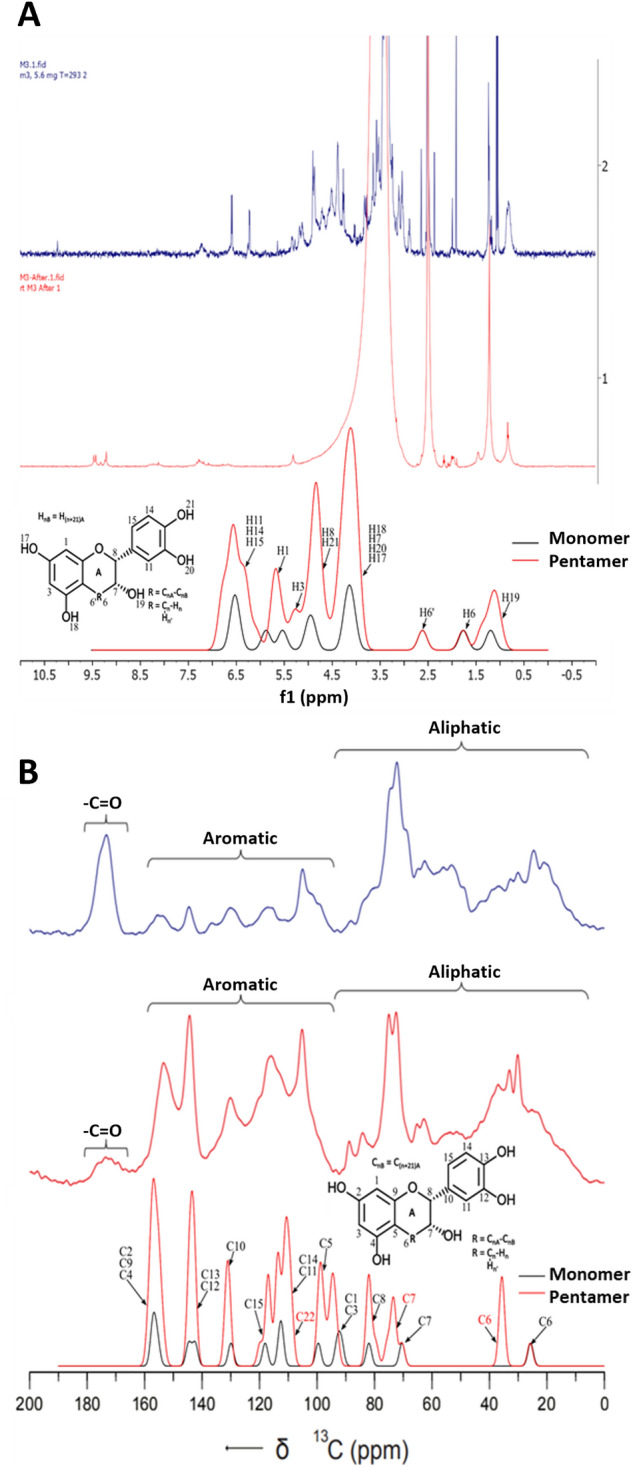
NMR chemical shifts of DM and corrected theoretical chemical shifts (gaussian broadened spectra) obtained for (−)-epicatechin monomer and pentamer in relation to experimental data (**A**) Solution state ^1^H-NMR chemical shifts, (**B**) Solid state ^13^C-NMR chemical shifts.

The 1D ^1^H NMR spectrum of the DM dissolved in deuterated DMSO (Fig. 6A) exhibited broad signals in the aromatic region (6–8 ppm) typical for oligomeric proanthocyanidins. The signals between 3 and 5 ppm were interpreted as residual solvent (DMSO and H_2_O), which were broader in the acid-treated melanin samples^36^. Figure 6B shows the ^13^C CP/MAS NMR spectra of the DM before and after acid treatment. These spectra were well resolved and primarily revealed peaks from the aliphatic (0–92 ppm) and aromatic (100–160 ppm) regions. The spectra of the melanin samples obtained after the acid treatment revealed well-resolved resonances between 0 to 180 ppm with fingerprint regions characteristic of oligomeric proanthocyanidins^37^. The intense peaks centered at 31 and 74 ppm may be assigned to the C4 and C3 aliphatic carbons in the (−)-epicatechin subunits, respectively. Peaks appearing at around 84 and 89 ppm could be assigned to the *cis* and *trans* C2 carbon atoms in the aliphatic ring, respectively. The peaks appearing between 100 to 160 ppm could be assigned to the different aromatic carbons in the flavan-3-ol units^38^.

The high-frequency NMR peaks, appearing at approximately 154 ppm, could be assigned to C5, C7 and C9 of the aromatic A-ring and the peak appearing at around 144 ppm may be attributed to the C3′ and C4′ of the B-ring. The peaks appearing at around 130 and 116 ppm may be assigned to the C1′ and C5′ of the B-ring sites respectively. However, the spectrum obtained after acid treatment revealed reduced intensity for carboxylic resonance at 173 ppm compared with the spectrum before acid treatment, which consistent with the FTIR spectra in the region with reduced C=O absorption. As mentioned above, there is a possibility that acid treatment breaks C=O linkages causing depolymerization of lignin and hemicellulose^39^. A comparative analysis between the experimental and theoretical chemical shifts supported the presence of (−)-epicatechin units in the samples.

**Figure 7:**
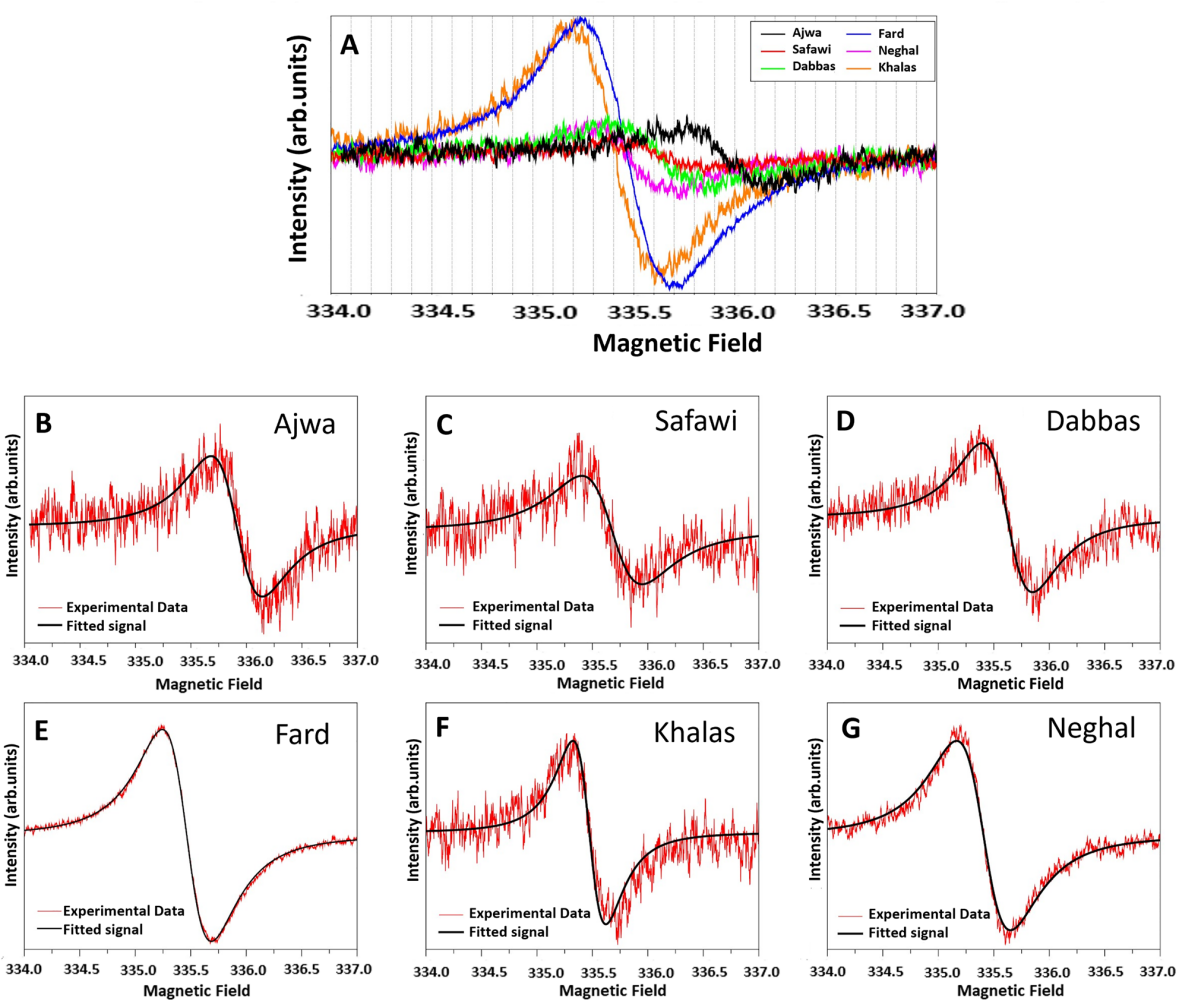
Electron paramagnetic resonance (EPR) spectrum of melanin extracted from six cultivars (**A**) experimental data, and (**B**–**G**) fitting of experimental (red lines) with Lorentzian-simulated spectra (black lines).

The EPR spectra determined experimentally and the simulated spectra of the melanin extracted from six cultivars, presented in Fig. 7A and B, respectively. For simulation, initially balanced Gaussian and Lorentzian curves were considered but Lorentzian portion provided excellent fitting. The incorporation of hyperfine, and g-strain did not affect the fittings. The EPR spectra of the DM showed strong resonance absorption, consisting of single, symmetrical lines devoid of hyperfine splitting. Their profiles appeared almost identical to those exhibited by melanin derived from other sources such as sunflower seeds^40^, black oats^41^, black seeds^42^. EPR is a robust technique for examining the electron spin characteristics of materials, substantially contributing to the understanding of electronic structure and behavior of melanin paramagnetic species^43^. The *g* values provide information regarding the chemical environment of unpaired electronic spins in the material, thus enabling the identification of specific magnetic interactions and some features of the electronic structure of the compounds. Furthermore, the ΔHpp parameter provides information regarding unresolved hyperfine interactions and g-factor (and other) anisotropies, which are associated with the width of the spectral line observed in the EPR spectrum.

Table 1 presents the EPR parameters and simulation fitting data of six date fruit melanin. The *g* values obtained from the samples in the present study were similar to those reported previously for other natural melanin sources such as chestnut (2.0042–2.0043)^44^, mushroom (2.0050)^45^ and melanin monomer/dimers (2.004–2.006)^25^. The line width (ΔH_pp_) of the samples was found between 0.4 and 0.53 mT. The smaller (ΔHpp) values were observed for brown color cultivars Khalas (0.44 mT) and Neghal (0.4 mT). This may be due to the fact that protected free radicals in the extracted melanin are less susceptible to hyperfine and spin–spin interactions. Since samples of the same weight were used to record the EPR spectra, the variation in the intensities of the obtained signals results from the difference in the concentration of paramagnetic radicals between cultivars. Complete understanding of the EPR behavior of the DM will only be possible when the chemical structure of this melanin is fully revealed.

**Table 1:** Electron paramagnetic resonance (EPR) parameters of melanin extracted from six date fruit cultivars aligned with EPR fitting data.

Cultivars	*g* values*	Peak-to-peak linewidths (Lorentzian)	Density of spin (Spin g^−1^)	Δ H_PP_ (mT)	Intensity (a.u)
Ajwa	2.0037	0.463	19	0.53	1090
Safawi	2.0034	0.544	15	0.5	660
Dabbas	2.0038	0.455	39	0.5	1250
Fard	2.0047	0.442	60	0.5	4790
Khalas	2.005	0.486	15	0.44	4580
Neghal	2.0046	0.297	18	0.4	870

Microwave Freq: 9.4205 GHz for Ajwa* and 9.41255 GHz for the other samples.

**Figure 8:**
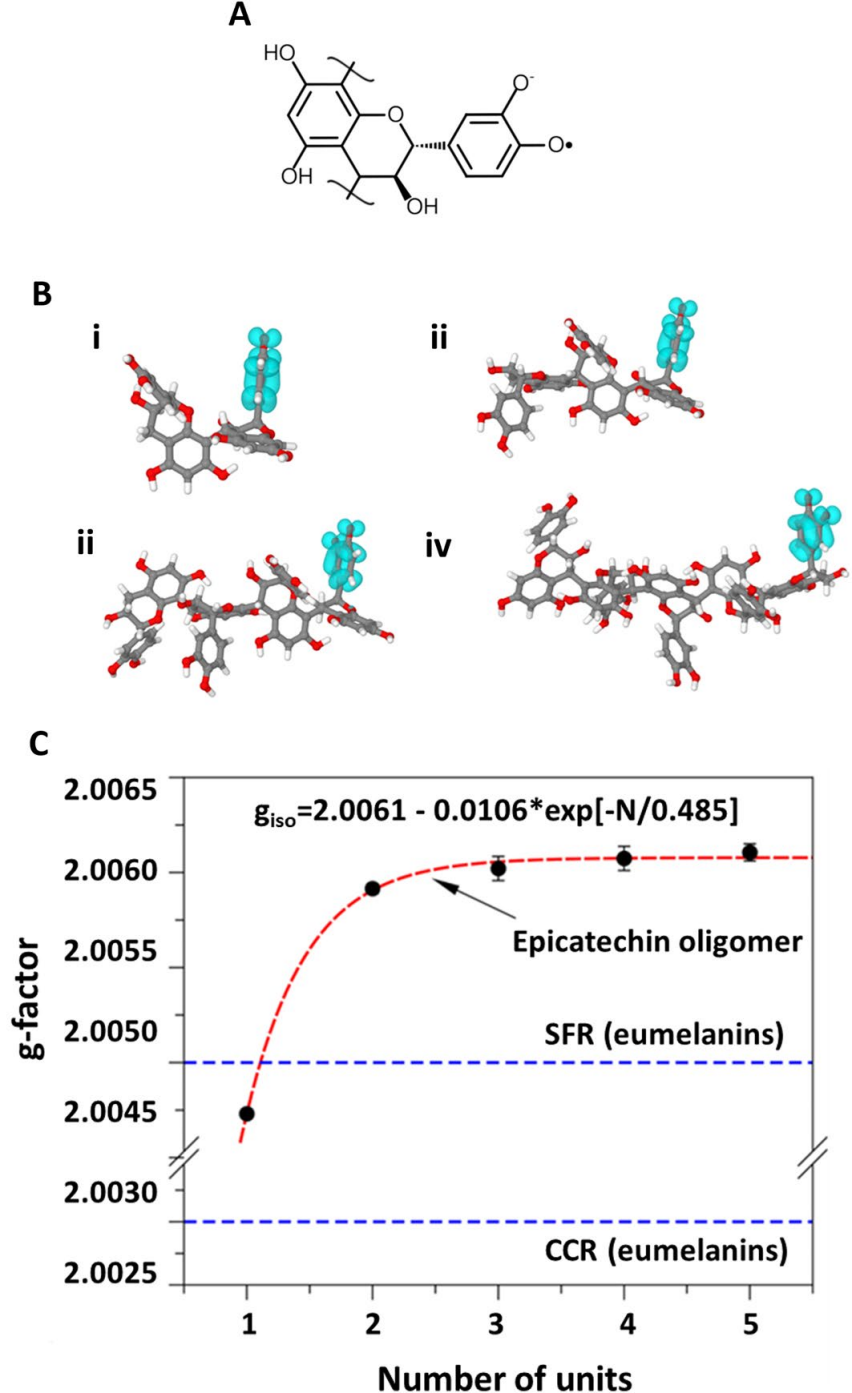
(**A**) Radical structure of (−)-epicatechin oligomer; (**B**) electron spin density distribution of (−)-epicatechin oligomers: (i) dimer, (ii) trimer, (iii) tetramer and (iv) pentamer, (**C**) Influence of the number of repeating units on the g-factor of (−)-epicatechin oligomers.

The persistent EPR signal of traditional eumelanin, either synthesized or extracted from animals, was associated with two distinct paramagnetic species, generically denominated carbon-centered-radicals (CCR) and semiquinone free radicals (SFR)^43^. CCR dominates the EPR signal in dried (and acidic) samples, resulting in lower g value (~ 2.004) and higher line widths, and are associated with intrinsic defects. On the other hand, SFR defines an extrinsic defect, which dominates the EPR response in aqueous dispersions (and alkaline samples) and exhibits higher g-factors and smaller ΔHpp values. The formation of SFR is promoted by electron exchange (comproportionation equilibrium) between partially oxidized units of melanin^46^. Figure 8 shows a comparative analysis of the g-factor estimated for (−)-epicatechin oligomers (SFR analog) relative to that of traditional eumelanin. The electron spin density distribution on (−)-epicatechin oligomers (only one representative structure) is also presented. The g-factor of the oligomers increases with the addition of (−)-epicatechin units, with saturation around 4–5 units (g ~ 2.0061). In general, the position of the radical in the oligomer had a small influence on the g-factor, with slightly reduced values for central radical units (Δg_iso_ ~ 10^–4^). The unpaired electron is primarily centered on the oxygen atoms of the units, similarly to the SFR of traditional eumelanin^47^.

## Conclusions

To the best of our knowledge, this is the first structural analysis of DM. Chromatographic and spectroscopic NMR and EPR studies, supported by DFT calculations, suggested that DM consists of agglomerated oligomeric (−)-epicatechin proanthocyanins. The aggregation behavior revealed via TEM and the insolubility of melanin in organic solvents suggests π–π stacking similar to that found in sepia ink melanin. Determination of the exact size of the oligomer(s) was challenging as the g-factor of EPR reached a steady state after 4–5 oligomeric units. The molecular weight of the solubilized DM was found to range 569–3236 Da, corresponding to 2–11 (−)-epicatechin monomeric units. The exact determination of the constituent oligomeric units requires MALDI-TOF mass spectrometry analysis. The discovery of the existence of melanin in date fruits and its characterization is expected to open a new area of research due to its significance in the nutritional and sensory characteristics of plant-based foods. Melanin can contribute to the color and the stringency of dry fruits such as dates and may affect their functionality in the gastrointestinal tract through their effects on free radicals and bacterial communities.

## Data Availability

All data generated or analyzed during this study are included in this published article.
