# Author Correction: Date fruit melanin is primarily based on (−)-epicatechin proanthocyanidin oligomers

**DOI:** 10.1038/s41598-024-57743-2

**Published:** 2024-03-26

**Authors:** Muneeba Zubair Alam, Clinton Emeka Okonkwo, João P. Cachaneski-Lopes, Carlos F. O. Graeff, Augusto Batagin-Neto, Saeed Tariq, Sabu Varghese, Matthew J. O’Connor, Abuzar E. Albadri, J. Beau W. Webber, Mohammed Tarique, Mutamed Ayyash, Afaf Kamal-Eldin

**Affiliations:** 1 Department of Food Science, College of Agriculture and Veterinary Medicine, United Arab Emirates University, P.O. Box: 15551, Al-Ain, United Arab Emirates; 2 Postgraduate Program in Materials Science and Technology (POSMAT), São Paulo State University (UNESP), Bauru, SP Brazil; 3 Department of Physics, School of Sciences, São Paulo State University (UNESP), Bauru, SP Brazil; 4 Institute of Sciences and Engineering, São Paulo State University (UNESP), Itapeva, SP Brazil; 5 Department of Anatomy, College of Medicine and Health Sciences, United Arab Emirates University, Al Ain, United Arab Emirates; 6 Core Technology Platforms, New York University Abu Dhabi, 129188 Abu Dhabi, United Arab Emirates; 7 Department of Chemistry, College of Science, Qassim University, 51452 Buraidah, Saudi Arabia; 8 Lab-Tools Ltd., Marlowe Innovation Centre, Marlowe Way, Ramsgate, CT12 6FA UK; 9 National Water and Energy Center (NWEC), United Arab Emirates University, P.O. Box: 15551, Al-Ain, United Arab Emirates

Correction to: *Scientific* *Reports* 10.1038/s41598-024-55467-x, published online 28 February 2024

The original version of this Article contained an error in the spelling of the author João P. Cachaneski-Lopes which was incorrectly given as João P. Cachaneski-Lope.

The original Article has been corrected.

